# Effects of earthquake spatial slip correlation on variability of tsunami potential energy and intensities

**DOI:** 10.1038/s41598-020-65412-3

**Published:** 2020-05-21

**Authors:** Jorge G. F. Crempien, Alejandro Urrutia, Roberto Benavente, Rodrigo Cienfuegos

**Affiliations:** 1 Pontificia Universidad Católica de Chile, Department of Structural and Geotechnical Engineering, Santiago, Chile; 2 Research Center for Integrated Disaster Risk Management (CIGIDEN), Santiago, Chile; 3 Universidad Católica de la Santísima Concepción, Department of Civil Engineering, Concepción, Chile; 4 Pontificia Universidad Católica de Chile, Department of Hydraulic and Environmental Engineering, Santiago, Chile

**Keywords:** Geophysics, Natural hazards, Seismology

## Abstract

Variability characterization of tsunami generation is quintessential for proper hazard estimation. For this purpose we isolate the variability which stems solely from earthquake spatial source complexity, by simulating tsunami inundation in the near-field with a simplified digital elevation model, using nonlinear shallow water equations. For earthquake rupture, we prescribe slip to have a log-normal probability distribution function and von Kármán correlation between each subfault pair, which we assume decreases with increasing euclidean distance between them. From the generated near-field inundation time-series, emanating from several thousand synthetic slip realizations across a magnitude 9 earthquake, we extract several tsunami intensity measures at the coast. Results show that all considered tsunami intensity measures and potential energy variability increase with increasing spatial slip correlations. Finally, we show that larger spatial slip correlations produce higher tsunami intensity measure exceedance probabilities within the near-field, which highlights the need to quantify the uncertainty of earthquake spatial slip correlation.

## Introduction

There are few well instrument recordings of destructive tsunami intensities produced by large subduction zone earthquakes, which makes the task to characterize tsunami hazard a very difficult one. With the developments of tsunami numerical modeling, there have been great advances in determining the actual hazard through the quantification of tsunami intensity measures (TIMs) using numerical modeling. Even with all the data available after an event, due to the epistemic uncertainty of the seismic source rupture parameters, there is a great variability of tsunami initial conditions (TIC), as well as tsunami inundation forecasting, as shown by many authors^1–3^. Hence, in order to better understand tsunami potential impacts, it is necessary to simulate many scenarios, with physics-based methods, to quantify the inherent TIM aleatory uncertainty, that is, uncertainty attributed only to the earthquake source variability, characterized by the different earthquake rupture processes which take place on faults^4^.

Two of the most important questions related to near-field tsunami intensity, for hazard assessments, are: How do TIM average and variability change with the variation of key earthquake source parameter statistics? How do the TIM variability control the annual rate of exceedance of TIMs, given a seismicity rate? To answer these questions, it is crucial to identify some of the key earthquake source parameters that can affect both the average and variability of the TIMs. The most important source parameter that can control TIMs is magnitude, since large earthquakes usually produce large tsunami waves and inundations. Therefore, in this study we simulate subduction earthquakes with a target moment magnitude M_w_9, which are very likely to produce large tsunami waves.

Another factor that largely controls TIMs is the coastal topo-bathymetry. However, in this study, we focus solely on the source contribution. Then, to isolate the impact of the spatial rupture process, we have designed a simplified topo-bathymetric digital elevation model (DEM), which includes only the first order features. In this way, we avoid unwanted coastal wave processes (which cannot be attributed to the earthquake source process) such as edge waves^5–7^, wave focusing on bays and submarine canyons^8–10^, resonance on the continental shelf^11–13^, among other effects. Other parameters such as geometry and size relative to the earthquake magnitude of the faulting area are of great importance^14^, which show an increase of peak near shore tsunami amplitude, which is primarily due to the peak slip areas within the fault. Increased ratios of seismic moment to area are shown to yield higher TIM amplitudes results with respect to homogeneous slip fault models as shown by An *et al*.^15^, provided the total rupture area is small. It is for this reason that the determination of earthquake scaling relationships between area and seismic moment are related to the TIM values.

To carry out the tsunami simulations, the fault extensions (i.e., length and width) need to be prescribed with proper earthquake scaling relationships. For these parameters, many relationships have been constrained from finite fault slip inversions of seismic data, which relate the earthquake size, width and length with respect to magnitude. The earliest work of this nature was accomplished for earthquakes with magnitudes ranging between M_w_ 4.7 and 8.2^16^. More recent scaling relationships have been derived for crustal and subduction zone earthquakes^17–22^.

Knowledge of the statistics of fault slip is vital for producing stochastic slip realizations that mimic expected behaviour of earthquakes. Using the SRCMOD database^23^, it has been found that slip distribution on faults can be characterized as a truncated exponential distribution^24^, which has a heavy-tail, allowing many more incursions into great slip extreme’s. Much earlier, other works have shown^25,26^ that for several crustal earthquakes, the slip distribution resembles more a truncated Cauchy distribution, which without any truncation is a self-similar distribution. Several authors use log-normal probability distributions to characterize slip on faults^27–30^. They simulate an underlying Gaussian random field by expanding a finite amount of Karhunen Loève (KL) terms^31^, which later, they take into the logarithmic domain with a resulting log-normal random field for slip. Log-normal slip distribution has also recently been employed as a prior probability distribution for seismic waveform slip inversion, within a Bayesian framework^32^. The log-normal distribution naturally accounts for slip positivity, not requiring truncation. In this study, we assume slip to be a log-normal distribution.

Owing to the vast amounts of evidence which shows that the earthquake rupture process is spatially complex, it is important to probe the effects fault spatial slip distribution have on tsunami wave generation. Much work has been done to measure the spatial correlation of slip, that is, the size of the slip asperities of an earthquake, with the available finite fault source inversions of seismic, geodetic and tsunami data. The spatial correlation has been quantified by several authors, including the seminal findings of Mai and Beroza^33^, who initially measured slip correlation by fitting several analytical solutions of power spectrum density (PSD) models to the power spectra of finite fault inversions. Most of the events in this catalog correspond to crustal earthquakes. For the case of subduction zone earthquakes, there are several studies which indicate that the slip PSD have a flat portion up to a corner wavenumber, after which the amplitude decays. Skarlatoudis *et al*.^20^ fit a PSD to a two dimensional Butterworth filter, Goda *et al*. and Raghukanth and Sangeetha^34,35^ fit a von Kármán (VK) PSD to large subduction zone earthquakes. All three studies show that the slip correlation lengths of these earthquakes scale with magnitude. In particular, the latter used the SRCMOD finite fault source database^23^ to constrain the correlation length parameter for each earthquake. These results show a radial correlation length of *L*_*c*_ ≈ 70 km for a magnitude *M*_*w*_9 earthquake. Melgar and Hayes^36^ have computed directly spatial correlations by fitting a VK auto-correlation in the spatial domain, estimating correlations between magnitude, along strike and down dip correlation lengths, effective lengths, etc. Results show a mean Hurst exponent of 0.36 and correlation lengths along strike and down dip of approximately *L*_*x*_ ≈ 150 and *L*_*z*_ ≈ 80 km for a magnitude *M*_*w*_9 earthquake.

The use of complex earthquake rupture distributions for tsunami simulation have been recently adopted in several studies. Among the first tsunami wave propagation simulations using complex rupture sources, we point out the work of Geist^14^, who introduced a seminal effort to determine the statistics of TIMs using a *k*^2^-model slip correlation consistent model^37^. He concludes that peak near-shore wave height can vary by a factor of three, with the inclusion of slip heterogeneity. Other studies^38^ use linear runup models and assumed a plane wave propagation to determine inundation. These assumptions produce large variability of runup intensities along the coast. Using low resolution DEMs, such as GEBCO^39^, Goda *et al*.^40^ have further shown that along shore runup variability increases dramatically with the inclusion of stochastic realizations of slip across faults. Furthermore, it has been shown, for the near field case of the Hikurangi subduction zone^41,42^, that peak runup amplitudes are much greater that the runups produced by equivalent magnitude homogeneous slip earthquake model. Fukutani *et al*.^43^, use a stochastic model to simulate kinematic earthquake rupture on M_w_9 faults on the same place where the 2011 M_w_9.1 Tohoku earthquake ruptured. They concentrate greater amount of seismic moment in the shallower portions of the subduction zone, concluding that the inclusion of slip heterogeneity on the fault increases the annual probability of exceedance of tsunami wave height, based on several stochastic realizations.

In the spirit of performing Probabilistic Tsunami Hazard Assessment (PTHA)^44–46^ type analyses, several studies have concentrated on wave height calculations using heterogeneous earthquake slip models, without any inundation calculations. LeVeque *et al*.^28^ incorporate earthquake source complexity in their tsunami wave propagation models, by incorporating several KL expansion terms, based on the assumption of a Gaussian random field. They later impose slip positivity by taking the logarithmic value of slip at each point of the fault domain. Sepúlveda *et al*.^29^ simulate the tsunami wave propagation of the 2014 M_w_ 8.2 Iquique earthquake by utilizing the KL expansion to characterize the earthquake source through stochastic slip simulations. Murphy *et al*.^47^ use dynamic rupture simulations to constrain physics-based compliant kinematic rupture of large subduction zone earthquakes. With their results they simulate tsunami wave propagation at near-field distances, for the particular case of the 2011 Tohoku earthquake. Li *et al*.^48^ use a *k*^2^ source model in the South China Sea, simplifying the tsunami propagation to the linear regime, so that, tsunami waveforms can be efficiently modeled as a superposition of Green’s functions. Sepúlveda *et al*.^30^ shows that with the inclusion of correlation length uncertainty, the hazard integral is unmodified, in comparison to the great sensitivity if has to the seismicity-rate quantified through the Gutenberg-Richter law. Their results are for the intermediate to far field shore locations and specifically for the South China Sea. It is worth noting that for far-field tsunamis, magnitude and directivity are critical source parameters^49,50^.

Tsunami wave generation calculated from heterogeneous earthquake slip models with high resolution DEMs to capture the runup and inundations processes have been performed initially by Mueller *et al*.^41^, with several stochastic realizations of earthquake slip on the Hikurangi subduction zone interface. They estimated TIMs such as inundation and flow depth. Since then, there have been many other similar contributions^2,51,52^.

In the last years, there have been several contributions regarding the estimation of structural fragility functions for tsunami damage assessment purposes with alternative TIMs, such as momentum flux, flow velocity and other related TIMs^53–55^.

In this work, we quantify TIM statistics due to distributions of different slip asperity sizes due to a M_w_9 earthquake scenario, i.e. we vary the VK radial correlation length of slip, in order to quantify TIM variability. To this end, we have implemented a stochastic earthquake rupture model which allows for changes in the degree of spatial correlations of slip on the fault with ease. With this method, we have computed sixty thousand earthquake rupture scenarios, ten thousand for each radial slip correlation lengths (i.e. equal correlation lengths along strike and down dip) ranging between 10 and 60 km. We must point out that the radial correlation lengths are lower than the 70 km correlation length proposed by Mai and Beroza^33^. The reason behind these values correspond to no TIM significant statistical changes beyond 50 km radial correlation length (this is also explained in Results and Methods section). For each rupture scenario, we model the vertical deformations of the seafloor with an analytic solution^56^, summing all the contributions of free surface deformations due to each rectangular homogeneous slipping subfault on an elastic homogeneous half space. With the deformation, we have quantified the sea-surface initial conditions of each of the many rupture models, which are then used to simulate the tsunami wave generation process with a finite volume numerical method that solves a nonlinear version of the shallow water waves equations. With these simulations we have calculated, on a very simple bathymetry, TIMs such as runup, wave height, wave momentum, and velocity flows. Details pertaining the model setup and the simulations are explained in the Methods section.

## Results

**Figure 1:**
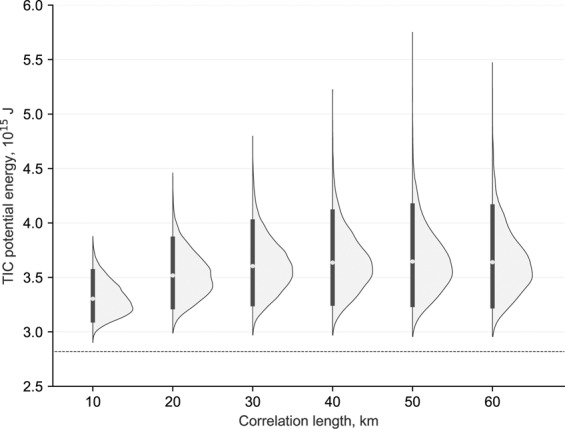
Probability density functions of the TIC potential energy for each correlation length. Thick black vertical line represents the 90% confidence interval and the gray circle represents the mean. The horizontal dashed line represents the TIC potential energy for an uniform slip.

The potential energy (*E*_p_) transferred by the earthquake rupture to the sea surface, is computed from the initial free surface deformation, *η*_*o*_^57^,

1 $${E}_{{\rm{P}}}=\frac{1}{2}\rho g{\iint }_{A}\,{\eta }_{o}^{2}dA,$$

where *ρ* is the seawater density and *g* is the gravity constant. The potential energy results obtained for each rupture scenario are shown in Fig. 1. Amongst these scenarios, we have included a uniform slip model, which always shows lower TIC potential energy than the heterogeneous models (a feature also shown by Melgar *et al*.^42^), which is always outside of the probability density distributions obtained from the stochastic finite fault slips. The average and variability of TIC potential energy increases with correlation length, and tend to saturate at the largest values. Because of the quadratic nature of potential energy shown in Eq. (1), the distributions show long-tail structures for higher values of potential energy as depicted in Fig. 1.

**Figure 2:**
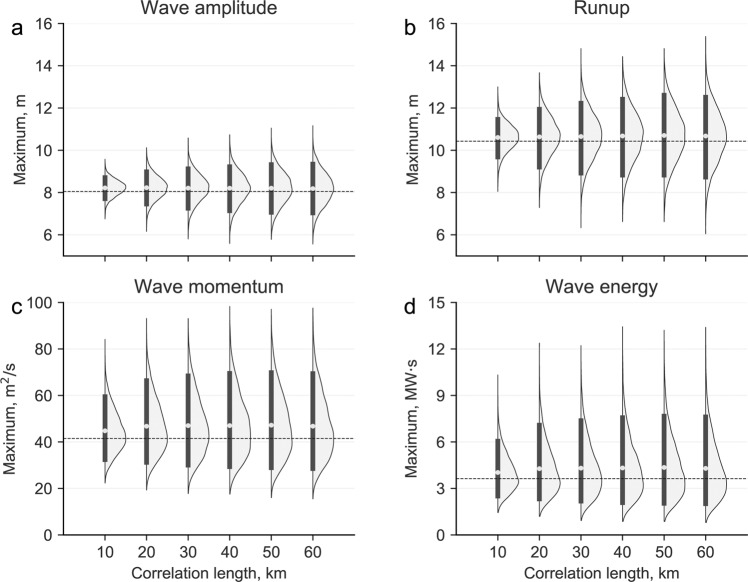
Probability density functions for the maximum values of (**a**) wave amplitude, (**b**) runup, (**c**) wave momentum and (**d**) wave energy, for each correlation length. Thick black vertical lines and gray circle represent the 90% confidence interval and the mean, respectively. The dashed line represents the results of the tsunami simulation with uniform slip.

**Figure 3:**
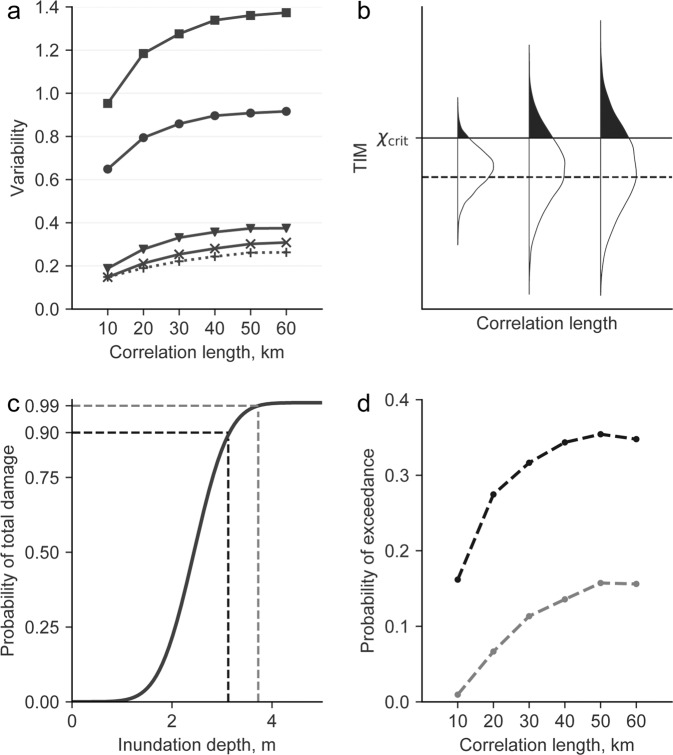
(**a**) Normalized variability for TIC potential energy (+), wave amplitude (×), runup (▼), wave momentum (•), and wave energy (▪). (**b**) Variability effect on the estimation of TIM probabilities of exceedence, which correspond to the shaded area above a critical TIM value, depicted as a continuous line. As a comparison, the dashed line depicts TIM from a uniform slip earthquake source. (**c**) Tsunami fragility curve for total structural damage of structures in terms of inundation depth, the two critical values are marked with dashed lines, at 3.2 m with a probability of 90%, and 3.7 m with a probability of 99%. (**d**) Probability to exceed each critical value, from which total structural damage, 3.2 m in black, and 3.7 m in gray.

In Fig. 2, we present the probability density functions for the maximum values of TIMs as a function of the VK slip correlation length. Mean values of the distributions are rather insensitive to changes in correlation lengths, and are slightly higher than the values obtained with a uniform slip. The effect that correlation length has on the probability density distributions is seen more clearly in the left panel of Fig. 3, where we compute the coefficient of variation by dividing the 90% confidence interval length by the mean value of the distribution. The coefficient of variation for all TIMs is larger as the correlation length increases, but it tends to saturate for correlation lengths above 50 km. The coefficient of variation is below 0.4 for TIC potential energy, wave amplitudes, and runup; among these, runup values show the largest variability, mostly controlled by nonlinear processes. Wave momentum and wave energy, show much higher variability coefficients, and a more pronounced dependence on the correlation lengths. Figure 3c,d depict asymmetric long tails towards higher values for wave momentum and wave energy respectively. Just as for the case of TIC tsunami potential energy, this is due to the quadratic nature of these TIMs, which can be seen directly in Eq. (13).

In the Supplementary Section, we show that 10000 simulations are enough to capture up to 99% confidence interval for all TIMs. Also, in this section we show that to capture the up to the 99% confidence interval, it is not necessary to do 10000 simulations, for some TIMs much less simulations are required.

## Discussion

We have implemented a slip model, based on observed earthquake characteristics, that preserves the imposed VK spatial correlations of slip on faults. When the VK correlation length increases, we observe an increase of the TIM variability. Uniform slip earthquake scenarios are widely used for PTHA calculations, however they are not able to capture the TIM variability which arise from the complexity of the rupture process. This has great implications on the PTHA integral proposed by Cornell^58^, where we can state that at any specific point in a coastline, the annual rate of exceedance for any TIM above a critical value of *χ*_crit_ will be:

2 $$\begin{array}{c}\lambda (TIM > {\chi }_{{\rm{crit}}})\,=\lambda (M > {m}_{{\rm{\min }}}){\int }_{{m}_{{\rm{\min }}}}^{{m}_{{\rm{\max }}}}\,P(TIM > {\chi }_{{\rm{crit}}}|m){f}_{M}(m)dm,\end{array}$$

where *λ*(*M* > *m*_min_) is the rate of occurrence of earthquakes greater than *m*_min_, rupturing at any portion of the pre-defined seismogenic zone, *f*_*M*_(*m*) is the probability density function of the magnitude distribution, and *P*(*TIM* > *χ*_crit_|*m*) is the conditional probability of TIM exceedance, given a specific magnitude. Since the mean TIM values are constant for any correlation length, the probability exceedance for *χ*_crit_ values greater than the mean TIM should increase with greater correlation lengths, as shown in 3. Since uniform slip models have no variability, the probability exceedance of this specific model will be smaller for extreme values, therefore the annual rate of TIM exceedance will also increase for extreme values of *χ*_crit_, when considering greater VK slip correlation lengths. Although these results have been calculated assuming M_w_9 earthquakes, they should hold for other magnitudes based on the assumptions of earthquake source self-similarity we have made^21,42,59^. To illustrate the consequences of slip correlation length has on the tsunami risk of coastal infrastructure, we use the tsunami fragility curve (Fig. 3c) developed by Aránguiz *et al*.^60^. This total damage fragility curve was developed using post-tsunami survey data collected after the 2015 M_w_8.4 Illapel earthquake, with no distinction of structural typology (e.g. wooden, masonry or steel structures). From the curve, we determine the inundation depths corresponding to 90 and 99% probability of total damage, which are 3.2 and 3.7 m respectively. With these inundation depths, we estimate the probability to exceed these critical values, for each slip correlation length. These results are shown in Fig. 3d, presenting the same tendency to increase the probability of exceedance as the correlation lengths become greater. These findings will have great impact on the newly derived energy and moment flux fragility functions constrained by^53–55^.

Beyond 50 km of VK slip correlation length, all TIM variability values tend to saturate, due to the finite amount of available TIC potential energy, which are later radiated into tsunami waves. Since correlation length determined the average size of the slip asperities, and therefor of the larger seafloor deformation areas, there is a point at which the fixed dimension of the fault geometry cannot contain larger slip asperities. Another constraint in our model that hinders on the average asperity growth is the potency scaling relation imposed on each slip model. We expect no TIM saturation when the fault geometry increases for different earthquake magnitude scenarios.

Our simulations are meant to explore TIM variability with respect to to correlation length, however there are several key earthquake source parameters which have great uncertainties and will have an impact on TIM variability as well. Among some of these parameters we can mention the Hurst parameter, magnitude scaling relationships for rupture area, length and width, fault geometry, etc. Also, it is important to mention that the current spatial slip correlation models^34–36^ are constrained with global finite fault models from different subduction regions. With the future increase of finite fault models, in the future it may be possible to determine characteristic spatial slip correlations for each subduction region, which will improve TIM variability estimations.

As a final observation, inundation will depend on the source location, local DEM and near field effects, like subsidence. However, the inundation of a uniform slip scenario will be in average, lower than the heterogeneous distributions^42^, but still higher than ruptures with slip concentrated in the deeper portions of the subduction interface. Although the simplified bathymetry that we present is capable to generate complex hydrodynamic processes such as edge waves, in conjunction with spatially complex earthquake rupture patterns^5,6^, it obscures other hydrodynamic processes that are known to have important controls on tsunami propagation and inundation (and hence on TIMs). Among them shelf and local resonance, which are important to describe spatial and temporal variations of tsunami hydrodynamics when acting over complex realistic bathymetries^3,12,13,61^. At this stage, it is not possible to predict the effects of spatial rupture complexities have on TIM variability when considering real bathymetries, due to the non-linearity of these complex hydrodynamic processes and local reflections that may induce wave amplification. To answer this question it is necessary to perform different numerical experiments of non-linear inundations on realistic topo-bathymetries.

## Methods

**Figure 4:**
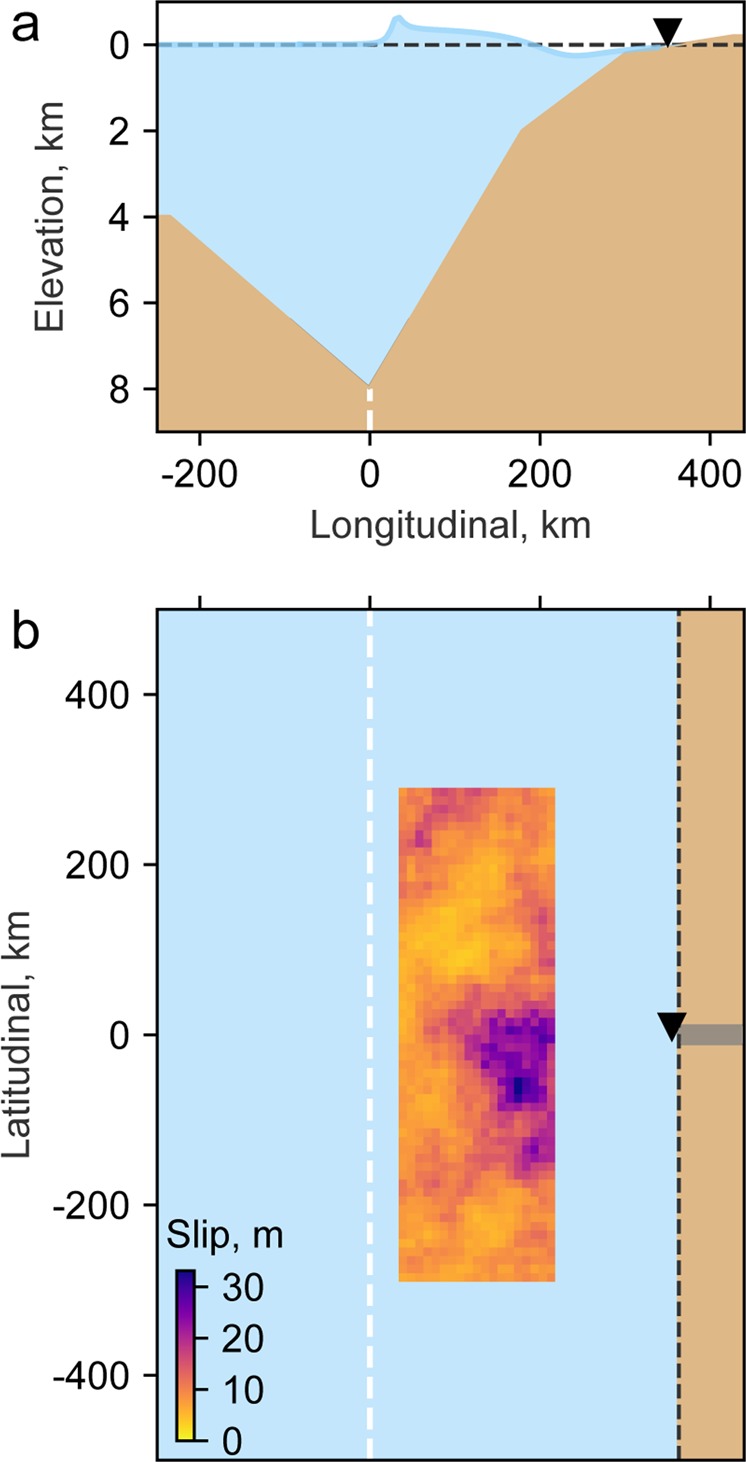
(**a**) Example of a TIC drawn with a vertical exaggeration over a simplified digital elevation model with a constant geometry along latitude. (**b**) Slip model seen from above, the dashed white line represents the synthetic trench, while the dashed black line represents the shoreline. The black triangle in the coast shows where the TIMs are calculated, with the exception of runup which is calculated in the gray zone. Lighter shades shown in the fault represent areas with lower amount of slip.

A stochastic slip distribution is generated to obtain finite fault scenarios of slip across a M_w_9 target earthquake, which is later used to propagate tsunami waves on a simplified topo-bathymetry, as shown in Fig. 4. The inundation is obtained from the tsunami wave propagation process for several thousand earthquake scenarios. To this end, we have constructed a method based on the enforcement of spatial slip correlations in the spatial domain using a VK compatible radial slip spatial correlation. Other subfault parameters are not taken into consideration, such as rupture velocity, rise-time and peak-time, which translates into assuming an instantaneous rupture across the fault.

### Stochastic slip generation model

The model we have constructed is only valid for subduction mega-earthquakes, for magnitudes greater than M_w_7.5. Given a Moment Magnitude as a target, we use the subduction interface scaling relationship of effective fault area, length *L* and width *W* of Allen and Hayes^21^, which are shown below,

3 $$\begin{array}{l}\log \,L=-2.90+0.63\cdot {{\rm{M}}}_{{\rm{w}}}\\ \log \,W=-0.86+0.35\cdot {{\rm{M}}}_{{\rm{w}}}\end{array}$$

The uncertainty of these scaling relationships is quite large (with standard deviation between 0.3–0.4), a property that most definitely should increase the variability of TIMs, and can be explored more thoroughly in the future.

Following recent works^27–29^, we have assumed log-normal probability distributions for the slip on target faults. For the spatial correlations of slip, we use an autocorrelation function consistent with the VK PSD, used in the work of Mai and Beroza^33^. The seismic rupture scenarios we generate have correlations lengths of 10 to 60 km. Since we fix the moment magnitude to a target value, we obtain the seismic moment (M_0_) through the following relation^62^:

4 $${{\rm{M}}}_{{\rm{w}}}=\frac{2}{3}(\log \,{M}_{0}-9.05)\mathrm{}.$$

Given a target moment magnitude ${{\rm{M}}}_{{\rm{w}}}^{{\rm{T}}}$, we compute the target seismic moment ${{\rm{M}}}_{0}^{{\rm{T}}}$. Assuming that our slip samples average uniformly distributes such a seismic moment across *N* subfaults, we have:

5 $${\bar{{\rm{m}}}}_{0}=\frac{{{\rm{M}}}_{0}^{{\rm{T}}}}{N},$$

where ${\bar{{\rm{m}}}}_{0}$ is average moment for every subfault. Then, the average slip vector $\bar{{\bf{s}}}$ is given by:

6 $${\bar{s}}_{i}=\frac{{\bar{{\rm{m}}}}_{0}}{{G}_{i}{A}_{i}},$$

where *G*_*i*_, and *A*_*i*_ are the shear modulus, and area of the *i* th subfault, respectively. The area *A*_*i*_ is equal to 10 × 10 km^2^. We assume that the logarithm of slip vector *x* follows a multivariate normal distribution, with mean *μ* and covariance matrix ∑:

7 $$\begin{array}{c}{\mathscr{N}}(\mu ,\Sigma )={(2\pi )}^{-\frac{N}{2}}det{(\Sigma )}^{-\frac{1}{2}}\\ \,\times \,\exp \left(-,\frac{1}{2},{({\bf{x}}-\mu )}^{{\rm{T}}},{\Sigma }^{-1},(,{\bf{x}},-,\mu ,)\right).\end{array}$$

Therefore, slip samples *s*_*i*_ follow a log-normal distribution. We let the mean of such distribution be $\bar{{\bf{s}}}$ and decompose its covariance matrix **C** into variances *ρ*^2^ and correlations **R**, such that:

8 $${C}_{ij}={\rho }_{i}^{2}{R}_{ij}.$$

Note that ${\rho }_{i}^{2}$ corresponds to the marginal slip variance of the *i*-th subfault. The slip correlation matrix **R** is constructed so that correlations for each subfault pair decays with distance according to a VK distribution^33,63,64^. VK type spatial correlations, for the slip field, have shown to describe reasonably the statistics of seismic rupture models obtained by finite fault inversions^33^ and are employed for stochastic slip simulation^27,29^. Then, we write the slip correlation matrix as:

9 $${R}_{ij}=\frac{{({r}_{ij}/{L}_{c})}^{H}{K}_{H}({r}_{ij}/{L}_{c})}{{2}^{H-1}\Gamma (H)},$$

where *L*_c_ is the correlation length, *r*_*ij*_ is the euclidean distance between subfaults *i* and *j*, *H* is the Hurst parameter, *K*_*H*_ is the modified Bessel function of the second kind for real order *H*, and Γ is the Gamma function. Following Žerovnik^65^, the covariance matrices for slip **C** and logarithmic slip ∑ are related as follows:

10 $${\varSigma }_{ij}=\,{\rm{l}}{\rm{n}}\left(1,+,\frac{{C}_{ij}}{{\bar{s}}_{i}{\bar{s}}_{j}}\right).$$

We can now relate our target mean slip $\bar{{\bf{s}}}$ to the log-slip mean using standard log-normal distribution statistics:

11 $${\mu }_{i}=\,\mathrm{ln}\,{\bar{s}}_{i}-{\varSigma }_{ii}^{2}\mathrm{/2}.$$

Thus, to obtain samples of the slip vector *s*, we simply exponentiate samples drawn from ${\mathscr{N}}(\mu ,\Sigma )$, where ∑ and *μ* are computed from Eqs. 10 and 11, respectively.

To sample the correlated normal distribution ${\mathscr{N}}(\mu ,\Sigma )$, we multiply the inverse of the Cholesky decomposition of ∑ with a vector of samples from a standardized uncorrelated normal distribution^32^. This approach allows for a more flexible specification of the covariance matrix, as opposed to the **KL** expansion method employed in previous works^27,29^, which require explicit calculation of the expansion terms for the desired covariance. Also, it is important to mention that this method allows the possibility to change the subfaults marginal variances, by specifying *ρ*^2^. This can be useful to resolve non-stationary spatial correlation structures of the rupture process, e.g., variation with depth or segmentation along strike of slip. Additionally, slip distribution samples are selected so that they satisfy the relation between the maximum ***P***_max_ and average potency ***P***_avg_ shown by Hayes^59^, within a margin of 0.15, which roughly corresponds to 1.5*σ* of his regression. Thus, we only retain samples that verify:

12 $$|\log \,{P}_{{\rm{\max }}}-(1.15\cdot \,\log \,{P}_{{\rm{avg}}}-0.87)| < 0.15,$$

where the potency for each *i*-th subfault is ${P}_{i}={s}_{i}\cdot {A}_{i}$. After simulating 60000 earthquake rupture scenarios, we obtain peak-slip values between 22 and 48 m.

### Tsunami model

To perform the tsunami simulations, we use the computational package, GeoClaw, which solves the nonlinear shallow water equations with a shock-capturing finite volume method^66^.

The TIC is obtained by first finding the deformed field of the seabed using the Okada solution^56^ produced by each realization of the earthquake slip model. The free surface field is assumed to be equal to the seabed deformation, and the rupture process to be instantaneous. Then, the tsunami is propagated over a simplified bathymetry with a trench far enough from the coast to avoid unwanted inland coseismic deformation. The inundation is modeled by allowing cells to dynamically change from dry to wet conditions, incorporating a bottom friction term^67^, therefore, we obtain runup, i.e. elevation above sea level at the point of maximum inundation. Finally, as shown in Fig. 4a, the inundation is simulated over a terrain with a smooth and constant slope, and a Manning roughness friction coefficient of 0.025 s/m^1/3^. In Fig. 4b we highlight in a gray shade the area where inundations are calculated.

### Tsunami inundation simulations

For the earthquake source, we use a Hurst parameter value of 0.75 as proposed by Mai and Beroza^33^, based on the finite fault database they collected, and a constant marginal slip standard deviation *ρ*_*i*_, equal to 5 m, however this value is selected to produce more realizations than the ones we use to simulate tsunami wave propagation, with the intention to select rupture scenarios that do satisfy the scaling relationship between maximum and average potency^59^. As mentioned before, we have simulated 10000 earthquake rupture scenarios for 6 different correlation lengths ranging between 10 and 60 km. The range we have selected for correlation lengths is in response to several preliminary simulations for correlation lengths of 90 and 120 km, which produce almost identical TIM variability results as the 60 km correlation length. This correlation length range is below the average value of a Mai and Beroza^33^ M_w_9 earthquake radial correlation length, which is ~70 km. As reference, inundations produced by a uniform slip scenario with the same magnitude M_w_9 are also considered. As shown in Fig. 4a, the top portion of the earthquake sources are located approximately 300 km away from the observation point depicted with an inverted black triangle, with the top edge buried at 8 km depth to avoid large shallow influences in our TIM results^51^; therefore the earthquake rupture simulations produce the required TIMs from the source area up to the inundated area.

The tsunami wave propagation and inundation is conducted over a simplified DEM to avoid alongshore effects on the hydrodynamics, with resolutions varying between 1000 to 5 m grid spacing, in 4 consecutive nested grids; the analysis is thus solely focused on the seismic source control. The target magnitude for these simulations is M_w_9, so we restrict our stochastic sampling to earthquake sources of magnitudes ranging between M_w_9 ± 0.01. The subfault spacing is 10 × 10 km^2^ and the shear modulus is 30 GPa. From the simulations, we obtain the TIMs of wave momentum (*M*_*x*_) and (*E*_*x*_) wave energy^68^. These quantities are computed at the center of the domain, at a location where the still water depth is 1 m. The mathematical expressions read respectively,

13 $$\begin{array}{rcl}{M}_{x} & = & h{u}^{2}\\ {E}_{x} & = & \rho hu(g\eta +({u}^{2}+{v}^{2})/2),\end{array}$$

where *u* and *v* are the velocity components in two Cartesian directions, *h* is the total water depth and *η* is the water level elevation above a datum. The wave amplitudes are measured at the same location. The maximum runup above the still water level is also extracted for each simulation.

## Supplementary information

Supplementary Information.
