# Supplementary material for: Effects of earthquake spatial slip correlation on variability of tsunami potential energy and intensities
Source: Sci Rep. 2020 May 21;10:8399. doi: 10.1038/s41598-020-65412-3 (PMC7242370; doi:10.1038/s41598-020-65412-3)
Supplement: Supplementary file 1 — Supplementary Information. [file 41598_2020_65412_MOESM1_ESM.pdf]

Supplementary section of "Effects of earthquake spatial slip correlation on variability of tsunami potential energy and intensities"

Jorge G. F. Crempien[1,2,*], Alejandro Urrutia[2], Roberto Benavente[3,2], and Rodrigo Cienfuegos[4,2]

[1]Pontificia Universidad Católica de Chile, Department of Structural and Geotechnical Engineering, Santiago, Chile
[2]Centro de Investigación para la Gestión Integrada de Desastres Naturales (CIGIDEN), Santiago, Chile
[3]Universidad Católica de la Santísima Concepción, Department of Civil Engineering, Concepción, Chile
[4]Pontificia Universidad Católica de Chile, Department of Hydraulic and Environmental Engineering, Santiago, Chile
[*]jocrempiend@ing.puc.cl

ABSTRACT

Spatial slip correlation model

For a 1-dimensional continous stochastic process $s(x)$, such that $x \in \mathbb{R}$, the autocorrelation (AFF) function can be defined for any pair $x, y \in \mathbb{R}$ as:

$$R_{ss}(x,y) = \langle s(x)s(y) \rangle , \tag{1}$$

where $\langle \cdot \rangle$ is the expected value operator. There are several analytic ACFs that are stationary, i.e. the ACF depends solely on the lag τ, and not on the position of observation x. Some of the most typical analytic stationary ACFs are the Gaussian and exponential models, which are equal to $R_{ss} = \exp(-\tau^2)$ and $R_{ss} = \exp(-\tau)$ respectively. To model 2-dimensional spatial slip across faults as stochastic processes we define as a von Kármán (VK) ACF[1–3], as:

$$R_{ss}(\tau) = \frac{G_H(\tau)}{G_H(0)}, \tag{2}$$

where H is the Hurst exponent, $G_H(\tau) = (\tau)^H K_H(\tau)$, with $K_H(\tau)$ being the modified Bessel function of the second kind, of a fractional order H, which we assume to be $H = 0.75$. τ is the autocorrelation lag between any two points on the fault, such that:

$$\tau = \sqrt{\Delta x^2 / L_{\mathrm{x}}^2 + \Delta z^2 / L_{\mathrm{z}}^2}. \tag{3}$$

This last expression is in terms of the along-strike and down-dip distances (Δx and Δx) and correlation lengths, L_{x} and L_{z} respectively, which make slip asperities larger in one direction with respect to the other (almost always larger along-strike direction). In this work we assume a stationary homogeneous correlation structure on the fault, which only depends on the euclidean distance between any two points on the fault , such that $r = \sqrt{\Delta x^2 + \Delta z^2}$, which translates into enforcing equality of the correlation lengths in both directions $L_{\mathrm{x}} = L_{\mathrm{z}} = L_{\mathrm{c}}$, and making the radial autocorrelation lag equal to $\tau = r/L_{\mathrm{c}}$. The denominator in equation 2 is equal to $G_H(0) = 2^{H-1}\Gamma(H)$, where $\Gamma(H)$ is the Gamma function. Therefore, given a distance of r between any two points on the fault that belong to a lattice (x_i, z_i) and (x_j, z_j), the radial VK correlation on the fault can be expressed in indicial notation as:

$$R_{ij} = \frac{(r_{ij}/L_{\mathrm{c}})^H K_H(r_{ij}/L_{\mathrm{c}})}{2^{H-1}\Gamma(H)}, \tag{4}$$

which is the expression that we used to constrain the covariance matrix $\mathbf{C}$, such that

$$C_{ij} = \rho_i^2 R_{ij}. \tag{5}$$

ρ_i^2 corresponds to the marginal slip variance of the i-th subfault. In Figure S1 we show 4 simulations with different stochastic seeds in each column, for different VK correlation lengths, spanning values between 10 and 60 km.

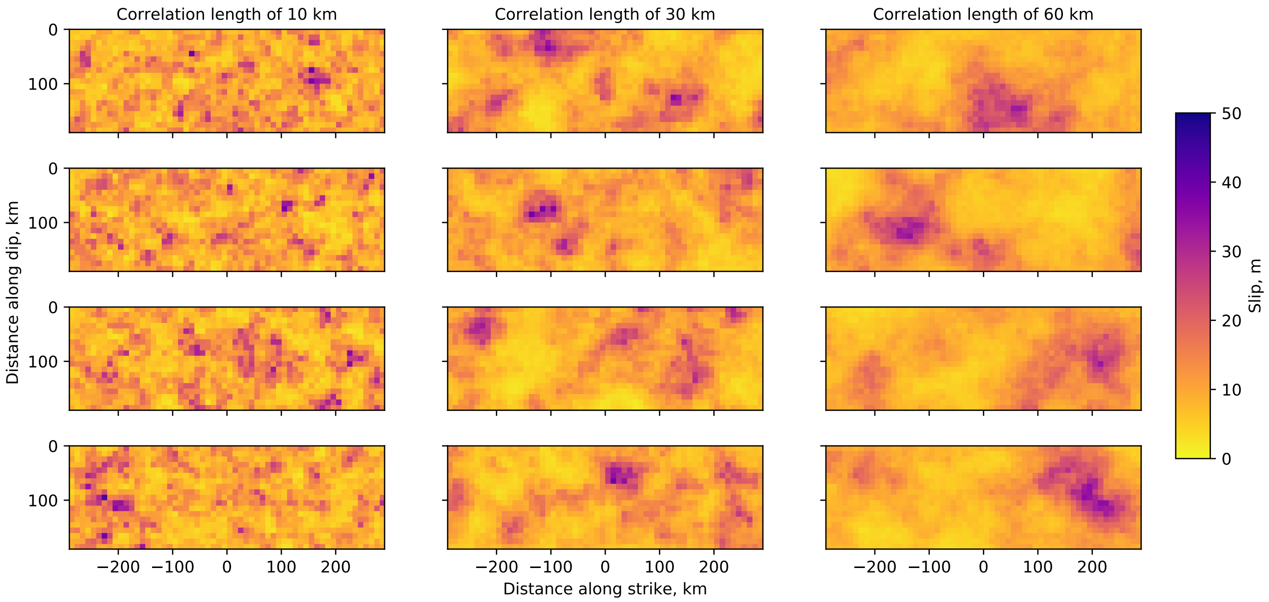

Figure S1. Simulations of magnitude 9 earthquakes with 4 different seeds and correlation lengths of $L_c = 10$, $L_c = 30$ and $L_c = 60$ km for a radially homogeneous VK spatial correlation structure.

Convergence of tsunami intensity measure confidence intervals

In Figures S2, S3, S4, and S5, we show the convergence confidence intervals of wave amplitude, runup, wave momentum and wave energy respectively. For each TIM, we simulate $10,000$ tsunami inundations, per correlation length for 50% (blue), 90% (orange) and 99% (green) confidence intervals.

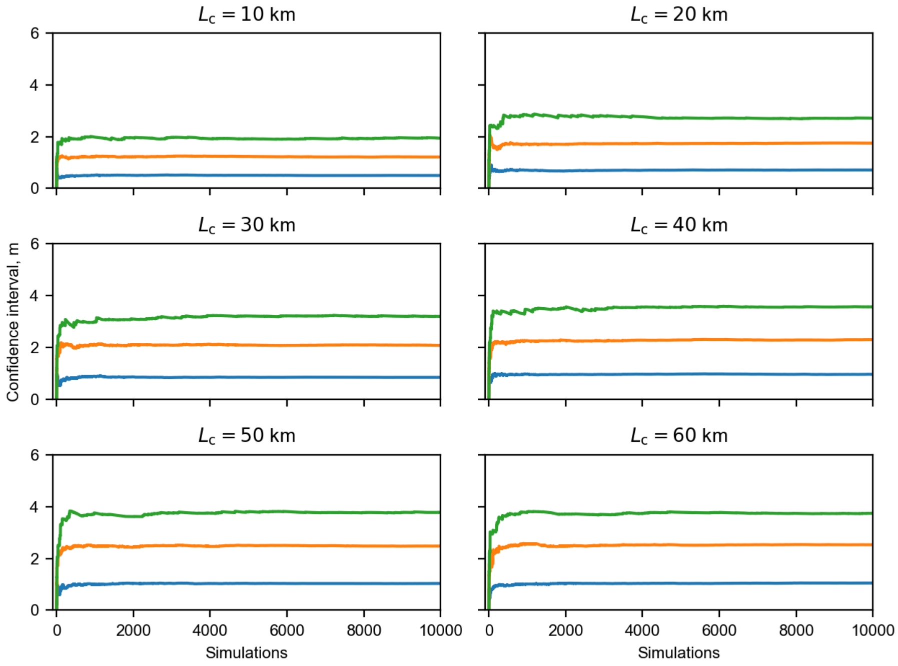

Figure S2. Convergence of wave amplitude confidence intervals of 50% (blue), 90% (orange) and 99% (green) for each correlation length.

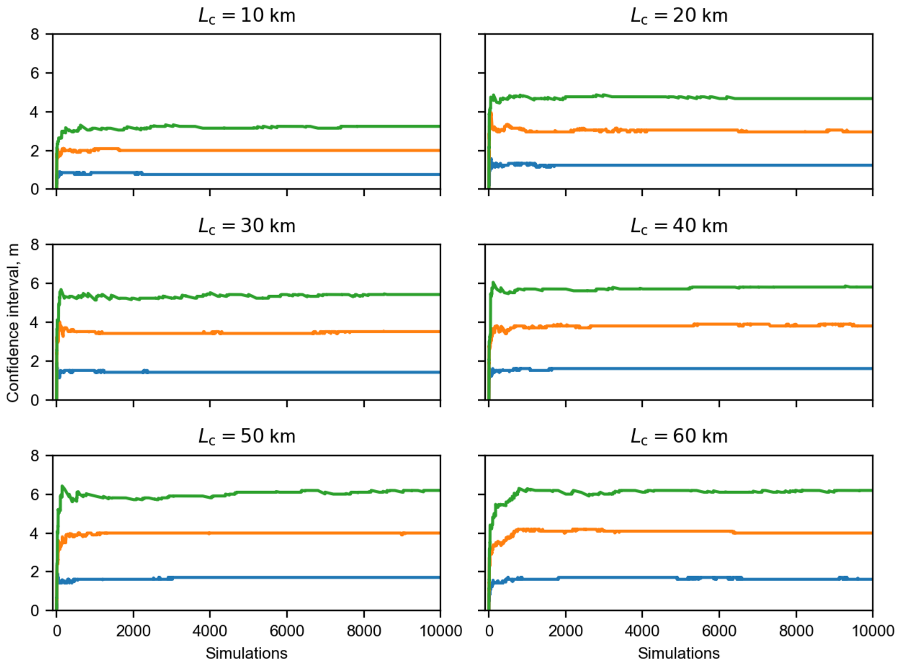

Figure S3. Convergence of runup confidence intervals of 50% (blue), 90% (orange) and 99% (green) for each correlation length.

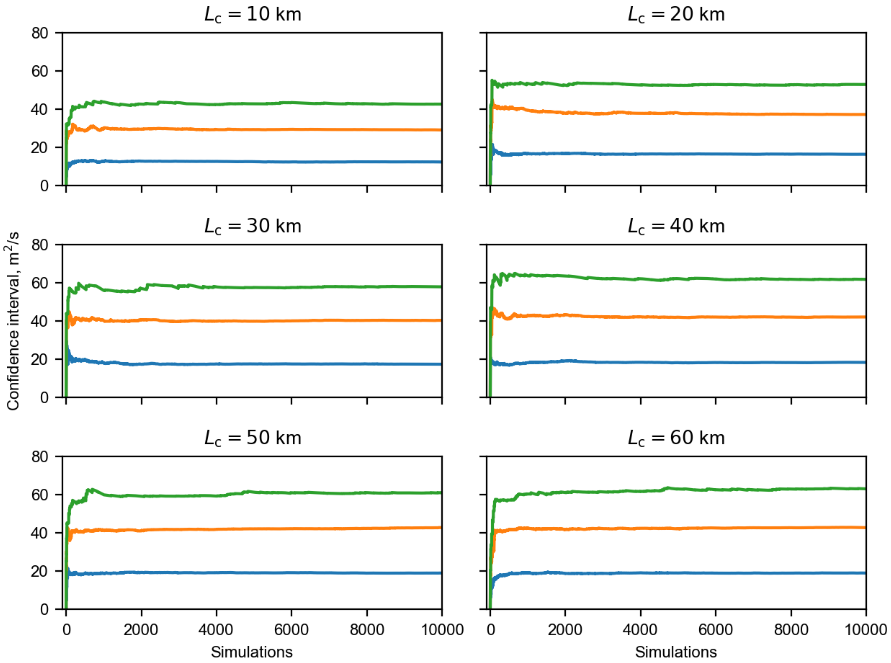

Figure S4. Convergence of wave momentum confidence intervals of 50% (blue), 90% (orange) and 99% (green) for each correlation length.

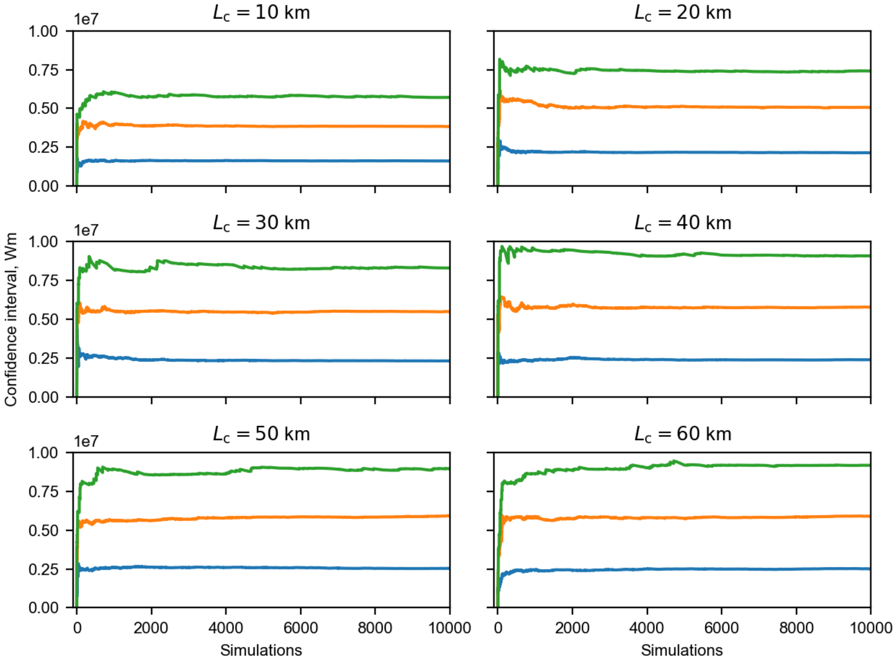

Figure S5. Convergence of wave energy confidence intervals of 50% (blue), 90% (orange) and 99% (green) for each correlation length.

References

1. Goff, J. A. & Jordan, T. H. Stochastic modeling of seafloor morphology: Inversion of sea beam data for second-order statistics. *J. Geophys. Res. Solid Earth* **93**, 13589–13608 (1988).
2. Mai, P. M. & Beroza, G. C. A spatial random field model to characterize complexity in earthquake slip. *J. Geophys. Res. Solid Earth* **107**, ESE–10 (2002).
3. Carpentier, S. & Roy-Chowdhury, K. Underestimation of scale lengths in stochastic fields and their seismic response: a quantification exercise. *Geophys. J. Int.* **169**, 547–562 (2007).
